# Tetrahydrocannabivarin (THCV) Dose Dependently Blocks or Substitutes for Tetrahydrocannabinol (THC) in a Drug Discrimination Task in Rats

**DOI:** 10.3390/biom15091329

**Published:** 2025-09-18

**Authors:** Hakan Kayir, Larissa Kouroukis, Iman Aziz, Jibran Younis Khokhar

**Affiliations:** Department of Anatomy and Cell Biology, Schulich School of Medicine and Dentistry, Western University, London, ON N6A 5C1, Canada; hkayir@uwo.ca (H.K.); lkouroukis2028@dents.uwo.ca (L.K.); imanaziz24@rcsi.com (I.A.)

**Keywords:** drug discrimination, Delta-9-Tetrahydrocannabivarin, THCV, cannabinoids, substitution, partial agonist, cannabinoid receptors

## Abstract

Delta-9-Tetrahydrocannabivarin (THCV), a naturally occurring cannabinoid and structural analog of THC, exhibits a dual pharmacological profile as a CB1 receptor agonist/antagonist and a partial CB2 agonist. This study evaluated the effects of THCV in a THC discrimination model in rats. Male Sprague-Dawley rats (*n* = 16, 300–340 g, PND60) were trained under a fixed ratio 20 (FR20) schedule to discriminate THC (3 mg/kg) from vehicle. Substitution tests were conducted with THC (0.325–3 mg/kg), THCV (0.75–6 mg/kg), and THC-THCV combinations. THCV produced an inverted U-shaped substitution curve, significantly differing from vehicle (*p* = 0.008). At 3 mg/kg, THCV partially substituted for THC (54.6% ± 17.82, *p* = 0.003). Response rate significantly increased during the substitution test with 3 mg/kg of THCV (*p* = 0.042). THCV (6 mg/kg) reversed THC (0.75 mg/kg)-induced responding (*p* = 0.040), with no significant change in response rate (*p* = 0.247). However, THCV combined with THC (1.5 mg/kg) affected response rates (*p* = 0.012), with 6 mg/kg significantly reducing rates vs. 3 mg/kg (*p* = 0.013). Blood THC and 11-OH-THC levels remained unchanged when THC was combined with THCV. The findings suggest THCV can partially mimic or block THC’s discriminative effects in a dose-dependent manner, possibly acting as a partial CB1 agonist.

## 1. Introduction

Cannabinoids, a diverse group of compounds found in the plant *Cannabis sativa*, have garnered significant scientific interest due to their wide-ranging effects on the human body. Cannabis contains over 550 chemical compounds, with more than 100 phytocannabinoids identified in the plant. Among these, ∆-9-tetrahydrocannabinol (THC) is the prominent psychoactive component of cannabis, and it has been extensively studied for its psychotropic and therapeutic effects [1].

Cannabinoids exert their effects by interacting with the endocannabinoid system, a retrograde signaling system comprising cannabinoid receptors, endogenous ligands, and enzymes [2]. The primary cannabinoid receptors, CB1 and CB2, are G-protein coupled receptors and widely distributed throughout the central nervous system and peripheral tissues, respectively. These receptors play a crucial role in modulating various physiological processes, including pain perception, mood regulation, and immune responses [3]. CB1 receptors are mainly located at presynaptic membranes of excitatory and inhibitory neurons, and they can inhibit voltage-gated Ca^2+^ channels and the release of GABA or glutamate. Thus, endocannabinoids or external substances with CB1 activity act as inhibitory retrograde neuromodulators [4].

Delta-9-Tetrahydrocannabivarin (THCV) is one of the cannabinoids extracted from cannabis and a naturally occurring structural analog of THC. THCV is a C_19_ propyl-tailed analog of THC, although it is derived from cannabidivarin, a different precursor molecule than THC [5]. In the plant, THCV is an inactive molecule in the carboxylic acid form that can be decarboxylated and activated when heated, dried, or exposed to light [6]. Unlike THC, THCV is generally considered non-psychoactive at low to moderate doses and cannot be discriminated from placebo in healthy humans [7]. On the other hand, a recent placebo-controlled study investigating the acute effects of THCV in healthy participants reported that THCV produced mild THC-like subjective effects, such as feeling and liking the drug, at higher oral doses (100–200 mg) [8]. THCV can induce tetrad effects (hypolocomotion, analgesia, catalepsy, and antinociception) in mice with a lower potency compared to THC [9]. THCV has appetite-suppressing properties which could aid in the management of obesity and is sometimes known as “Diet Weed”. THCV was also proven to reduce fasting plasma glucose levels compared against a placebo group, which could pertain to type-2-diabetes management [10,11].

THCV exhibits a complex pharmacological profile, functioning both as a CB1 receptor neutral antagonist in vivo at low doses and as a CB1 receptor agonist in vivo at higher doses [12]. In vitro, THCV behaves as a neutral antagonist rather than an inverse agonist [12]. THCV can mimic THC by inducing catalepsy and analgesia in mice and humans, although with lower potency. It also has hypothermic, hypolocomotive, and anxiolytic effects in mice [9]. It was initially thought to be a competitive CB1 agonist due to its ability to displace selective CB1 receptor agonists in mouse brain membranes. However, at lower doses, THCV can antagonize THC-induced effects in mice, a behavior also observed in in vitro assays [12]. Moreover, it produces a weak partial agonist response in the CB1 receptor cyclic adenosine monophosphate (AMP) inhibition assay [9]. Additionally, as a partial agonist at CB2 receptors, THCV inhibits forskolin-induced stimulation of cyclic AMP production in hCB2-CHO cells [12]. Moreover, THCV may exert indirect agonism at CB1 and CB2 receptors by increasing endocannabinoid tone as it can inhibit the anandamide transporter, which is responsible for carrying endocannabinoids to catabolic enzymes, and can also suppress the major endocannabinoid catabolic enzymes, fatty acid amide hydrolase, and monoacylglycerol lipase [10,13].

Drug discrimination is a well-studied, robust, and valid model for understanding the in vivo effects and testing the subjective effects of the drugs. It is particularly useful as a tool for drugs with multiple receptor activity at different doses such as THC [14]. Historically, THC and cannabinoids are some of the most studied substances in this assay [15]. The discriminative stimulus effects of THC in rats correspond well with the subjective effects experienced by humans, supporting the model’s validity. The pharmacological specificity of THC was proved by the inability of many other psychoactive drugs, including LSD, PCP, and psilocybin, to mimic THC effects in this model [16].

Previously, THCV was tested for THC discrimination only in a few studies. In a randomized, double-blind, placebo-controlled study, 10 male cannabis users were administered THCV (5 mg) or identical placebo capsules before administration of intravenous THC (1 mg) [7]. They could not distinguish THCV from placebo and still reported THC effect after THCV pretreatment. However, THCV pretreatment protected THC-induced impairments in a verbal recall test but increased the intrusions (recalling the words that were not related to part of the original list). In a recent study investigating the effects of cannabinoids on THC discrimination, THCV (3–30 mg/kg) did not substitute for THC in male and female Long Evans rats, and it slightly decreased THC-associated responding when combined with THC [17].

In the present study, we aimed to test the effects of THCV in a THC discrimination model in Sprague-Dawley rats. Furthermore, the blood levels of THC and its active metabolite 11-OH-THC were evaluated to rule out any possible pharmacokinetic interaction.

## 2. Materials and Methods

### 2.1. Animals and Housing

All procedures complied with the guidelines described in the Guide to the Care and Use of Experimental Animals (Canadian Council on Animal Care, 1993) and approved by the Animal Care Committee at the University of Guelph. Male Sprague-Dawley rats (Charles River, Raleigh, NC, USA), weighing 300–340 g and aged postnatal day 60 (PND56) upon arrival in the laboratory, were pair housed in the SealSafe^®^ Plus (Tecniplast, Varese, Italy) vent-rack cage with ad libitum access to food and water. They were maintained under a 12 h light/dark cycle (lights on at 7:00 a.m.) with constant ambient temperature (22 ± 2 °C) and humidity (50–70%). Behavioral tests were performed during the light phase of the light/dark cycle between 2 and 4 p.m. All the rats were handled daily for three days before starting the experiments. Five days prior to training, rats were placed on a food-restricted schedule in which they were allowed a limited amount of food to maintain approximately 85% of their free-feeding weight. Food was then restricted to approximately 10 g/day, in addition to the amounts obtained during training sessions on weekdays and was freely available on weekends (Friday afternoon to Sunday morning).

### 2.2. Drug Discrimination Procedure

All training and testing procedures were conducted in eight standard operant chambers placed in HABITEST (model: H10-24) isolation cubicles (Coulbourn Instruments, Allentown, PA, USA). The chambers contained a house light, a pellet dispenser attached to a food magazine equipped with a magazine light, and two retractable levers on either side of the magazine. Each isolation cubicle was equipped with an exhaust fan to provide background noise and ventilation, and a video surveillance camera. The chambers were controlled, and the responses were recorded by Graphic State software (v.3.03). The reinforcing stimulus was 45 mg banana-flavored sucrose pellets (Bio-Serv, Flemington, NJ, USA, product #F0024).

The drug discrimination protocol was adapted from a previous paper [18]. The food-restricted rats (*n* = 16) were trained to obtain one pellet under a gradually increasing fixed ratio (FR) schedule. Once animals reliably responded to the FR20 schedule, they were trained to respond to one of the levers following administration of the training drug, THC (3 mg/kg), and to respond to the other lever for the vehicle administration. Both training conditions were conducted under the FR20 schedule. The position of the lever was assigned randomly within the group of animals to control for lever bias. Responses on the incorrect (not treatment-appropriate) lever reset the ratio requirement on the correct lever. The sessions lasted 30 min or until the rats received 100 pellets, whichever came first. The order of the THC and vehicle administration was assigned randomly, with the restriction that no drug would be administered in more than three consecutive sessions. The discrimination training continued until the rats met the substitution test criteria, which include selecting the treatment-associated lever on the first FR20 response and selecting treatment-associated lever ≥80% of the total responses for six consecutive sessions.

Substitution tests were performed with THC (0.325–3 mg/kg), THCV (0.75–6 mg/kg), and their combinations (THC 0.75 and 1.5 mg/kg with THCV 3 and 6 mg/kg). A test session in four rats with THCV at 9 mg/kg dose was performed but was not included in the study because the rats were unable to give any response. Between test sessions, animals were kept on the regular training protocol and required to maintain the test criteria on three consecutive sessions to be qualified for the next test session. Test sessions were terminated after 20 min, or after the first 20 responses on either lever. No pellet was administered as a consequence of lever pressing in the test sessions.

### 2.3. Drugs

THC (Toronto Research Chemicals, Toronto, ON, Canada) and THCV (Kare Chemical Technologies, Mississauga, ON, Canada) were diluted in ethanol at 100 mg/mL concentration. The desired amount of THC or THCV were collected in glass tubes, and the ethanol was evaporated under nitrogen gas. A precalculated amount of Tween 80 was added to the remaining drug and vigorously mixed until the drugs were transferred to the solution. Saline (0.9% NaCl) was slowly added to the mixture while stirring at a final concentration of 1 mg/mL drug in Tween 80–saline solution (1:19 ratio). In order to settle the foamy mixture, the prepared drugs were kept in a fridge overnight and used after reaching room temperature on the next day. Vehicle was prepared similarly except with the addition of THC or THCV. Both THC and THCV were administered intraperitoneally (ip). The training or the test sessions started 30 min after THC or vehicle injections. THCV was injected 10 min after the THC or vehicle injection during the combination or substitution tests.

### 2.4. Blood Collection

Blood samples were collected 30 min after the THC administration (at the times at which rats would be placed in operant chambers) at the end of drug discrimination trainings (chronic), after the last THC (3 mg/kg) + THCV (6 mg/kg) combination treatment, and after a month of washout period (acute). The hind legs of the rats were shaved before starting the exposures. They were placed on heated pads for 5 min, Vaseline was applied to the area matching the saphenous vein, and a 22G needle was used to puncture the vein. The blood drops were collected with a capillary blood collection tube with a maximum volume of 300 μL (Microvette CB300, Sarstedt, Nümbrecht, Germany). The samples were kept in ice and were centrifuged at 8000 RPM for 5 min. The supernatant serum was transferred to the cryovials and stored in a −80 °C freezer until analysis.

### 2.5. Serum THC and 11-OH-THC Quantification

As previously mentioned [19], reference standards of THC and 11-OH-THC and their deuterated internal standards THC-D3 and 11-OH-THC-D3 were purchased from Sigma-Aldrich Canada (Oakville, ON, Canada). Captiva enhanced matrix removal lipid (EMR-Lipid) 96-well plate (Agilent, Santa Clara, CA, USA) was used to extract THC and 11-OH THC from the samples [19]. Briefly, 250 μL of acetonitrile (acidified with 1% formic acid) was added to each well, then 50 μL of rat serum and 20 μL of internal standard solution were added. After the sample passed through under positive pressure at 3 psi, the extraction plate was washed with 150 μL of a mixture of water/acetonitrile (1:4, *v*:*v*) solution. The effluent was evaporated under nitrogen at 40 °C, and the residual was reconstituted with the mobile phase for subsequent LC-MS/MS analysis. Calibration standards (2–1000 ng/mL) and quality controls (3 ng/mL and 800 ng/mL) were prepared on the day of analysis by spiking standard working solutions into blank rat serum. The liquid chromatography separation was achieved on a Vanquish Flex UHPLC system (Thermo Scientific, Waltham, MA, USA) as reported before [19]. Five microliters of plasma extracts were injected and separated on an ACQUITY UPLC BEH C18 Column (1.7 µm, 2.1 mm × 50 mm; Wexford, Waters, Ireland) connected with a VanGuard UPLC BEH C18 Pre-Column (Wexford, Waters, Ireland). The auto sampler was kept at 4 °C and column temperature was at 35 °C. The mobile phase consisted of the following. A: 10 mM ammonium formate with 0.1% formic acid aqueous solution, and B: acetonitrile with 0.1% formic acid. The flow rate was 400 μL/min under a gradient mode. The gradient conditions were sustained as follows: mobile phase B linearly ramped up from 40% to 95% from 0.1 to 4 min, and maintained at 95% for 2 min, then ramped back to 40%. THC and 11-OH-THC were eluted at 4.6 and 3.5 min, respectively, with a total run time of 7 min. MS analysis was conducted with a Q Exactive Focus Orbitrap mass spectrometer (Thermo Scientific, Waltham, MA, USA) equipped with an Ion Max source in positive electrospray ionization (ESI) mode. The source conditions were optimized as the spray voltage of 3.5 kV, the capillary temperature of 300 °C, and aux gas heater temperature of 425 °C. Data were acquired and processed in parallel-reaction monitoring (PRM) mode using TraceFinder™ software (v. 4.1, Thermo Scientific, Waltham, MA, USA). In this PRM mode, protonated 11-OH-Δ9-THC ion (*m*/*z* 331.23) and Δ9-THC ions (*m*/*z* 315.23) were selected as precursors, then fragmented in the higher-energy C-trap dissociation (HCD) cell at collision energy of 20 eV for 11-OH-THC and 25 eV for THC. The resulting MS/MS product ions were detected in the Orbitrap at a resolution of 17,500 (FWHM at *m*/*z* of 200) with AGC target set at 1 × 10^5^. The most abundant fragments from the MS/MS spectra (*m*/*z* 313.22 for 11-OH-THC and *m*/*z* 193.12 for THC) were selected as the quantifying ions. Other specific fragments, *m*/*z* 193.12 for 11-OH-THC and *m*/*z* 259.17 for THC, were selected as the confirming ions. The resulting chromatograms were extracted and reconstructed with a mass accuracy of 5 ppm for quantification and confirmation. The optimized MS/MS compound parameters are summarized in Table 1.

### 2.6. Statistics

For each discrimination session, percentage of responses (±S.E.M.) on the drug lever and response rate (lever presses/s) were calculated. Percentage of THC responding and response rates were analyzed using one-way analysis of variance (ANOVA) followed by Dunnett’s test for post hoc analyses. ED_50_ values with 95% confidence intervals (CI) were calculated using linear regression analysis. If rats pressed the levers less than 20 times, their preference data were excluded from the study, but the response rate was included. The level of statistical significance was set at *p* < 0.05. Statistical analyses were performed using the JASP v.0.18.3 [20] and GraphPad Prism v.10.3.1 (GraphPad Software Inc., San Diego, CA, USA) software.

## 3. Results

**Figure 1:**
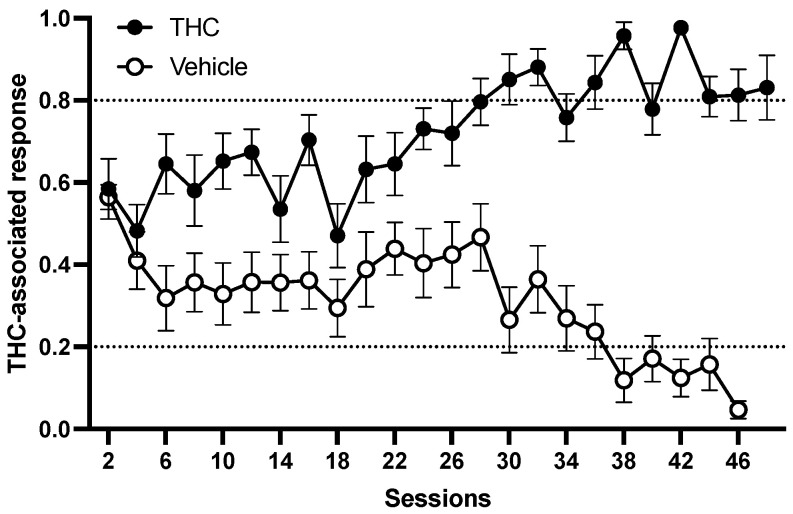
Acquisition of ∆-9-tetrahydrocannabinol (THC, 3 mg/kg vs. vehicle) discrimination.

**Figure 2:**
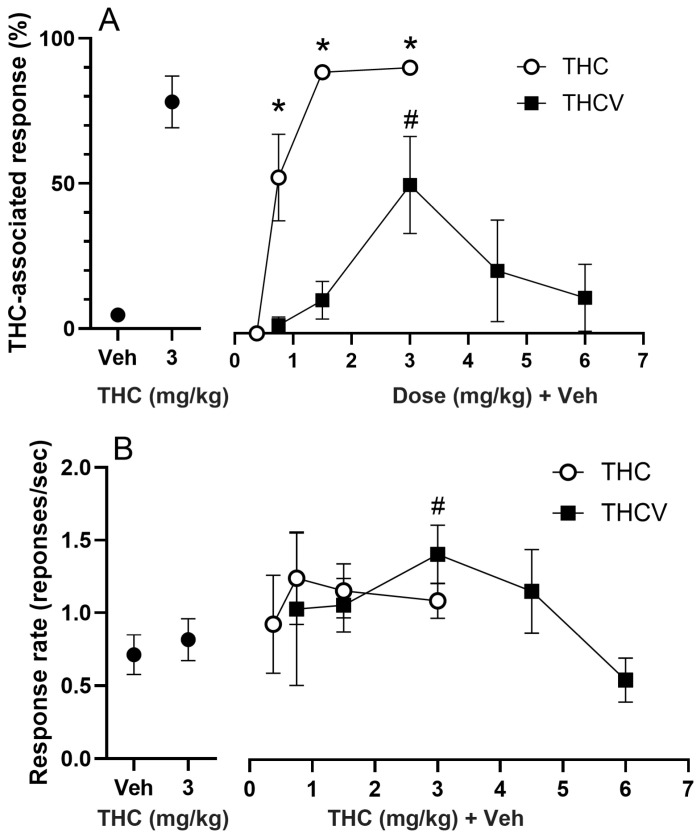
The ∆-9-tetrahydrocannabinol (THC)-associated lever presses at different doses of THC and ∆-9-tetrahydrocannabivarin (THCV) (**A**) and the response rate (**B**) of the rats. Averages of the vehicle (Veh) and THC training sessions during the tests are shown at the left side of the panels, and the results of the test sessions are given at the right side (* *p* < 0.05, THC vs. Veh, Tukey’s test; # *p* < 0.05, THCV vs. Veh, Dunnett’s test).

The average number of sessions needed to reach the criteria (correct response on the first FR20 schedule and ≥80% correct response for six consecutive sessions) is given in Figure 1. Thirteen rats among the sixteen rats trained to discriminate 3 mg/kg THC from vehicle learned the task in 50 ± 4 sessions. Three rats could not reach the criterion and were sacrificed after the 64th session. The rats showed full, dose-dependent substitution for THC at a training dose of 3 mg/kg [F(4,35) = 28.248, *p* < 0.001, η^2^ = 0.763, Figure 2A]. Post hoc tests indicated the rats could discriminate THC as low as 0.75 mg/kg dose (*p* = 0.002, Tukey’s test, Figure 2A). The ED_50_ for THC-associated discriminative stimulus effect for THC during the test sessions was 0.758 mg/kg (95% CI: 0.576–0.855 g/kg). The response rate during the training sessions and the substitution test did not show any significant alteration [F(4,32) = 1.141, *p* = 0.355, η^2^ = 0.125, Figure 2B].

The THCV substitution tests revealed an inverted U-shaped dose response curve that significantly differed from vehicle response [F(3,34) = 0.805; *p* = 0.008, η^2^ = 0.359, Figure 2A]. Post hoc Dunnett’s test showed that THCV at 3 mg/kg dose significantly deviated from Veh response, and it induced a partial (54.6% ± 17.82) THC-associated lever selection (*p* = 0.003, Figure 2A). Response rate of the rats was altered during the test sessions with THCV [F(5,31) = 2.698; *p* = 0.039, η^2^ = 0.303, Figure 2B]. Post hoc tests indicated that response rate significantly increased during the substitution test with 3 mg/kg of THCV (*p* = 0.042, Figure 2B).

**Figure 3:**
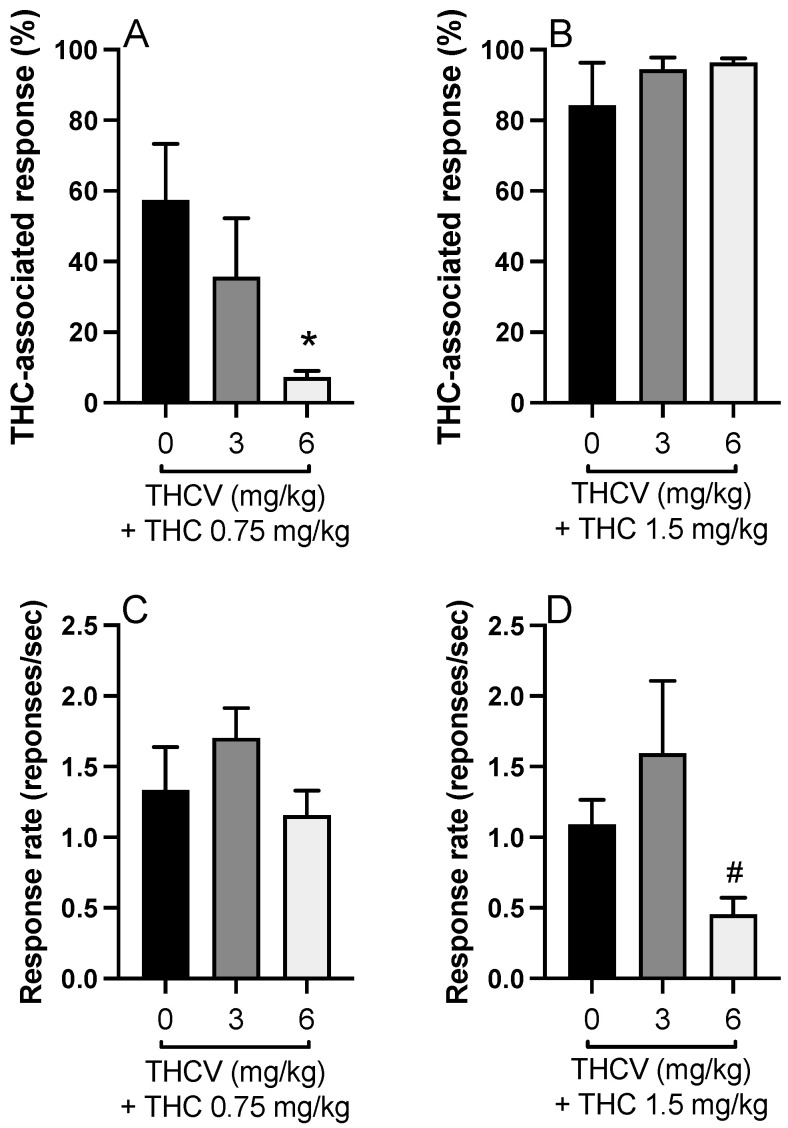
The ∆-9-tetrahydrocannabinol (THC)-associated lever presses of the rats during the combination treatment of ∆-9-tetrahydrocannabivarin THCV (3 and 6 mg/kg) with THC 0.75 mg/kg (**A**) and THC 1.5 mg/kg (**B**). Response rates of the rats during the combination treatment of THCV (3 and 6 mg/kg) with THC 0.75 mg/kg (**C**) and THC 1.5 mg/kg (**D**) (* *p* < 0.05, compared to THC alone, # *p* < 0.05, compared to THC 1.5 mg/kg + THCV 3 mg/kg, Tukey’s test).

Further, we combined THC (0.75 and 1.5 mg/kg) with THCV (3 and 6 mg). THCV at 6 mg/kg but not at 3 mg/kg reversed the THC (0.75 mg/kg)-associated responding [F(2,21) = 3.752, *p* = 0.040, η^2^ = 0.263, Figure 3A]. The response rate of the treatment groups was similar [F(2,20) = 1.499, *p* = 0.247, η^2^ = 0.130, Figure 3C]. On the other hand, combination of THCV at both doses did not create any effect on discriminative stimulus effect of THC at 1.5 mg/kg dose [F(2,17) = 0.665; *p* = 0.527, η^2^ = 0.073, Figure 3B], but the response rate was significantly affected [F(2,17) = 5.747, *p* = 0.012, η^2^= 0.403, Figure 3D]. Post hoc Tukey’s test indicated that THCV decreased the response rate in the discrimination test at 6 mg/kg dose compared to 3 mg/kg when it was combined with THC 1.5 mg/kg (*p* = 0.013, Figure 3D).

**Figure 4:**
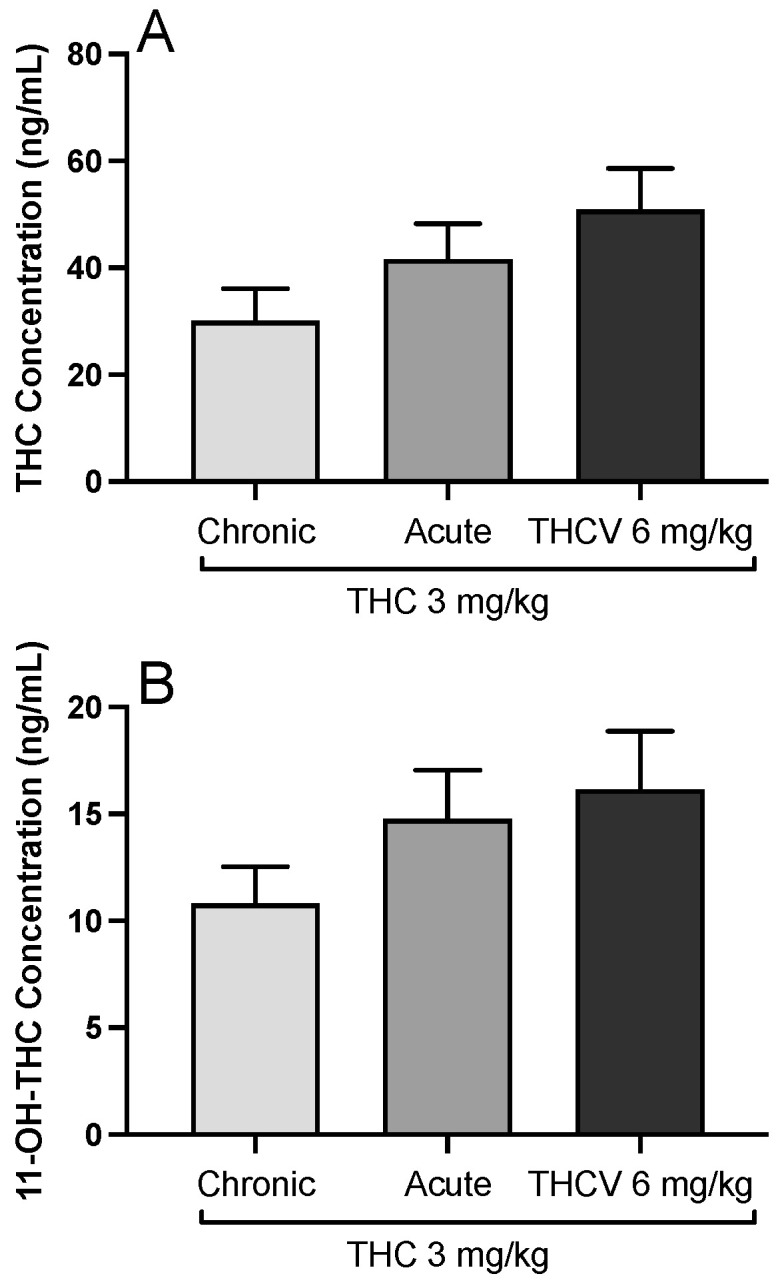
The blood levels of ∆-9-tetrahydrocannabinol (THC, (**A**)) and its active metabolite 11-OH-THC (**B**) in the rats during the trainings (chronic), after a single dose of 3 mg/kg THC (acute), and after a combination treatment of THC (3 mg/kg) and ∆-9-tetrahydrocannabivarin (THCV, 6 mg/kg).

A possible pharmacokinetic interaction between THC and THCV and the effect of chronic THC treatment during the trainings on THC metabolism was ruled out by blood concentration measurements of THC and its active metabolite 11-OH-THC. Neither THC [F(2,22) = 2.391; *p* = 0.115, η^2^ = 0.179, Figure 4A] nor 11-OH THC [F(2,22) = 1.836; *p* = 0.183, η^2^ = 0.143, Figure 4B] showed any change when THC was combined with THCV.

## 4. Discussion

The present study showed that THCV can block the discriminative stimulus effects of THC and partially substitute for THC in a dose-dependent manner. Thus, THCV may act as a partial agonist on the same receptors that THC produces its effects. Also, THCV increases the response rate at the dose it partially substitutes THC, which implies it can have stimulant properties. Finally, the observed effects were free from a possible interaction resulting in a change in blood levels of THC and its active metabolite 11-OH THC.

In vitro studies characterized THC as a partial agonist at both CB1 and CB2 receptors with moderate affinity and with no apparent receptor subtype selectivity. Thus, the activation of these target receptors will depend on the presence and the coupling efficiency of other ligands, reserve receptor density, and signal transduction efficiency. A wide variety of behavioral effects of THC is observed in vivo when other factors, such as biased agonism and endocannabinoid system regulatory activities, are considered. Similarly, THCV presents a complex pharmacological profile as indicated in numerous in vitro and in vivo assays [17,21,22,23]. In a previous review by McPartland et al. (2015) [6], THCV was suggested as a neutral antagonist of CB1 receptors and a partial agonist on CB2 receptors while it may show an indirect agonism at CB1 receptors by inhibiting endocannabinoid tone. A recent study confirmed that it has CB1 antagonist, but not agonist, effects in vitro [23]. However, there are studies implying THCV produces some in vivo activity through inverse agonism at CB1 receptors, and shows some opposing effects compared to other CB1 antagonists depending on the model, dose, and the availability of the other ligands [6]. Using the drug discrimination method provided a unique opportunity to compare the “interoceptive” effects of THCV and THC that represents a final behavioral outcome of the complex interplay between different interactions [24].

There are only a few studies that have tested THCV in animals trained to discriminate THC. In contrast to our results, a recent study by Moore et al. (2023) [17] indicated that THCV did not substantially alter THC’s discriminative stimulus effects across a dose range of 3–30 mg/kg, but it slightly decreased THC-associated responding at the higher doses in some cases, which the authors attributed to possible sedative effects rather than a direct modulation of THC’s interoceptive effects. We observed the sedative effects of THCV at 6 mg/kg dose when it was combined with THC 1.5 mg/kg. When it was given alone at the substitution experiments the response rate with the same dose (6 mg/kg) of THCV was not different from the control but there was a decreasing trend in lever pressing rate, and we did not try a higher than 6 mg/kg dose of THCV in these experiments.

In comparing our findings with those of Moore et al. (2023) [17], several key procedural differences may account for the contrasting results regarding THCV’s interaction with THC in a drug discrimination model. First, we performed our experiments in male Sprague-Dawley rats while Moore at al. used both male and female Long Evans rats. Our use of Sprague-Dawley rats, as opposed to the Long Evans rats employed in Moore et al., may have contributed to the observed differences, as strain-specific responses to cannabinoids have been documented in the literature. For instance, Deiana et al. (2007) [25] demonstrated that Long Evans and Lister Hooded rats, but not Sprague-Dawley rats, acquired and maintained self-administration of the cannabinoid CB1 receptor agonist WIN 55212-2, indicating significant strain differences in cannabinoid reinforcement behaviors.

Second, an important difference between the two studies was the timing of the drugs. Moore et al. administered THC 45 min before the session, followed by THCV (or another minor cannabinoid) given 15 min prior to the session. In contrast, in our study THC was administered 30 min before the session, while THCV was administered 20 min prior. This modified timing might create greater overlap in the peak activity of both compounds, allowing us to observe a dose-dependent partial substitution for THC by THCV at certain doses and an antagonistic effect at higher doses, where THCV reversed THC-associated responding.

In a study by Zagzoog et al. (2020) [9], the affinities of different cannabinoids were evaluated for the CB1 and CB2 receptors in order to further understand their pharmacodynamics. The compound CP55940, which has equal affinity to CB1 and CB2 binding, was used to test the characteristics of the cannabinoids. It was found that THCV partially displaced CP55940 from the CB1 receptor which indicated binding at an independent site on the receptor, or non-competitive binding. The same findings were concluded for the CB2 receptor as well. This aligns with our observation that THCV exhibits partial substitution effects at lower doses while antagonizing THC’s effects at higher doses. These findings emphasize the notion that THCV’s actions are modulated not only by direct receptor interactions but also by the presence of other ligands such as THC in the cannabinoid system components. Moreover, our findings support that THCV’s pharmacological effects are highly dose-dependent and influenced by complex receptor interactions, as seen in both in vitro studies and in vivo behavioral assays.

Our study also identified stimulant-like properties of THCV at doses where partial substitution for THC was observed, as evidenced by increased response rates. This finding challenges the conventional view of THCV as a cannabinoid devoid of psychoactive effects [10]. However, we did not perform additional tests specifically designed to assess psychostimulant activity, such as open field tests, locomotor activity assays, or detailed behavioral observations, to further explore this effect, and our claim is based solely on the observed increase in lever pressing rates during the test sessions. Another explanation for the increased response rate can be an anxiogenic or obsessive-like behavior exacerbation. This is consistent with a recent finding that THCV increased the number of marbles buried in a marble burying test [26]. Increased number of intrusions in a verbal recall task after a five-day dosing of THC and THCV combination in healthy cannabis users could indicate a possible pattern of obsessive or perseverative behavior [7]. Moreover, a recent placebo-controlled study investigating the acute effects of oral THCV treatment reported that euphoric mood was the most reported adverse effect [8]. Further studies employing similar dosing regimens or combinations with cannabis/THC in different human participants (i.e., healthy participants, chronic cannabis users) are essential to better understand the behavioral and physiological implications of these findings. Moreover, a better understanding of the effects of major and minor cannabinoids on cognitive processes could also clarify the increased response rates [27]. Lastly, combining these behavioral studies with measures of oscillatory activity could further inform our study of the neural basis of some of the perplexing behavioral interactions between THC and THCV [28].

In the present study, the observed behavioral effects are not due to an altered metabolism of THC or through its active metabolite 11-OH THC. The absence of such pharmacokinetic interactions is noteworthy given that cannabinoids often exhibit complex metabolic interactions, including competition for cytochrome P450 enzymes or modulation of drug-metabolizing enzymes [29]. This finding is consistent with previous reports that THCV does not significantly alter THC metabolism at the doses tested [7]. However, our results reflecting the peripheral blood concentrations do not rule out potential local interactions within the brain or other tissues. Regional differences in drug distribution or metabolism, particularly in areas dense in CB1 receptors, and receptor-level competition could still play a significant role in the observed behavioral outcomes [30].

While our findings advance our understanding of THCV’s discriminative effects, several limitations should be noted. First, our use of only male Sprague-Dawley rats greatly limits generalizability of our findings due to the previously documented strain- [25] and sex-dependent [19,31,32] effects of cannabinoids. Moreover, previous studies have demonstrated that male rats develop less tolerance to the effects of THC in the tetrad test. Moreover, anxiogenic effects were observed in female rats after THC treatment, whereas THC decreases anxiety in male rats [30]. The blood and brain concentrations of THC as well as its active metabolite 11-OH-THC were also found to be higher in female rats after edible THC consumption [19]. There is limited data on sex- and strain-dependent effects of THCV, but future studies focusing on these factors are warranted to determine whether similar patterns emerge for this compound. Second, we used food restrictions to maintain operant responding, and a sucrose pellet was used as the reward, both common practices in drug discrimination studies. Both THC and THCV have known effects on feeding and satiety. While THCV has been reported to suppress appetite [10], THC exhibits an orexigenic effect and increases food intake initially; however, tolerance to this effect may develop in rats after chronic treatment [33]. These interactions with feeding-related pathways could modulate motivational or interoceptive states and potentially influence discrimination performance independently of cannabinoid-like subjective effects. Finally, our study did not further evaluate whether the partial substitution of THCV could be reversed by CB1 or CB2 antagonists such as rimonabant or SR144528. These studies would be required to confirm that the observed effects are mediated by a cannabinoid receptor-mediated mechanism. However, since both THC and THCV display complex pharmacology, including partial agonist and dose-dependent effects at CB1 receptors, as well as activity at CB2 and non-cannabinoid targets, the antagonist combination studies would become particularly challenging to interpret. Moreover, the combinatorial outcome may depend not only on dose, but also on the relative efficacy, affinity, and dynamic balance between agonist and antagonist actions at multiple sites. While antagonist studies are an important future direction, the present findings provide a critical first step in establishing that THCV can partially substitute for THC in a drug discrimination paradigm.

## 5. Conclusions

Taken together, our findings highlight THCV’s unique pharmacological profile, characterized by partial agonism dose-dependent substitution for THC, and antagonism at higher doses. Importantly, THCV substituted for THC in a graded manner without evidence of pharmacokinetic interactions, and it also produced stimulant-like effects that distinguish it from THC. These results suggest that THCV may act as a dose-dependent modulator of cannabinoid receptor activity, capable of both mimicking and opposing THC’s discriminative stimulus effects. Such bidirectional properties are consistent with its complex receptor pharmacology and underscore the importance of dose in determining behavioral outcomes. Future studies should expand on these findings by examining sex- and strain-dependent variability, assessing the role of CB1 and CB2 receptor mechanisms using antagonist approaches, and exploring THCV’s actions across a broader range of behavioral paradigms, including those related to reward, cognition, and feeding behavior. Together, these efforts will help to clarify the pharmacology of THCV and further delineate its position within the cannabinoid spectrum.

## Figures and Tables

**Table 1:** Optimized LC-MS/MS compound parameters for quantitation of 11-OH-THC, CBD, and THC using PRM mode (CE: collision energy, *m*/*z*: mass/charge ratio, RT: retention time).

Analyte and Internal Standard	Precursor Ion (*m*/*z)*	CE	Quantitation Ion (*m*/*z)*	Confirming Ion (*m*/*z)*	RT (min)
11-OH-Δ9-THC	331.23	20	313.22	193.12	3.5
11-OH-Δ9-THC–D3	334.24	20	316.23	196.14	3.5
Δ9-THC	315.23	25	193.12	259.17	4.5
Δ9-THC–D3	318.25	25	196.14	262.19	4.5

## Data Availability

The original contributions presented in this study are included in the article. Further inquiries can be directed to the corresponding author.

## Abbreviations

The following abbreviations are used in this manuscript:

ANOVA	Analysis of Variance
Ca^2+^	Calcium Ion
CB1	Cannabinoid Receptor Type 1
CB2	Cannabinoid Receptor Type 2
cAMP	Cyclic Adenosine Monophosphate
CE	Collision Energy
CI	Confidence Interval
ED_50_	Effective Dose for 50% Response
ESI	Electrospray Ionization
FAAH	Fatty Acid Amide Hydrolase
FWHM	Full Width at Half Maximum
GABA	Gamma-Aminobutyric Acid
HCD	Higher-Energy Collisional Dissociation
ip	Intraperitoneal
LC-MS/MS	Liquid Chromatography-Tandem Mass Spectrometry
LSD	Lysergic Acid Diethylamide
MAGL	Monoacylglycerol Lipase
PCP	Phencyclidine
PND	Postnatal Day
PRM	Parallel Reaction Monitoring
RT	Retention Time
S.E.M.	Standard Error of the Mean
THC	∆-9-Tetrahydrocannabinol
THC-D3	Deuterated THC (Isotopically Labeled Internal Standard)
THCV	Tetrahydrocannabivarin
UHPLC	Ultra-High Performance Liquid Chromatography
η^2^	Eta Squared
